# Supplementary material for: A Bayesian Statistical Model Is Able to Predict Target-by-Target Selection Behaviour in a Human Foraging Task
Source: Vision (Basel). 2022 Nov 11;6(4):66. doi: 10.3390/vision6040066 (PMC9680426; doi:10.3390/vision6040066)
Supplement: Supplementary file 1 [file vision-06-00066-s001.zip › supplementary material part 1.html]

SVG: The Paper


# SVG: The Paper

#### Alasdair Clarke, Amelia Hunt & Anna Hughes

#### 14/10/2022

# 1 Trial Level Posterior Predictions

We will use the visual foraging data from Clarke (2022) to assess the model’s trial level performance. As the current version of our model only takes the \(p\_A\) bias into account for the initial target selection, we will ignore these data points for now.

```
# read in data
d <- read_csv("data/clarke_2020_qjep.csv", show_col_types = FALSE) %>%
  mutate(condition = as_factor(condition),
         condition = fct_recode(condition, feature = "1", conjunction = "2"),
         targ_type = as_factor(targ_type))
```

We will use the model fit as detailed in Clarke (2022). For simplicity’s sake, we will characterise all of our estimated posterior probability densities with just the mean value. This gives us four parameters per participant: \(b\_S, b\_A, b\_P, b\_D\).

Using these fitted values, we can now step through each target selection in the dataset and compute the probability weights our model assigns to each remaining target. For each target selection, we record:

- the item assigned the highest weight by our model.
- whether this item was then selected by the human participant.

```
# item weights pre-computed - see scripts/extract_item_weights_using_model_clarke.R
a <- readRDS("scratch/qjep_model_weights.rda") %>%
  filter(found != 1) # remove initial selections

# compute the average weight for each participant x condition x item selection
# also compute the proportion of times in which the item selected by the 
# participant was judged the most likely to be selected by our model.
a %>% group_by(condition, observer, found) %>%
  summarise(meanb = mean(b),
            prop_best = mean(selected_max), .groups = "drop") %>%
  # finally, also compute what we would expect under a null-model
  mutate(chance = 1/(41-found))-> a_agg
```

We can then plot this data to show how our ability to predict which target will be selected next varies throughout a trial. We can see a difference between *feature* and *conjunction* foraging, with *feature* foraging being more predictable (Figure 1).

Figure 1. (*left*) The average weight assigned to each selected target by our model. (*right*) The proportion of trials the item with the largest assigned weight was selected by the participant. Each dot shows data from an individual participant in a condition and the shaded region indicates the interval in which we expect 67% of participants to fall.

## 1.1 Calibration

Is our model calibrated? By this we mean, if our model assigns an item a probability \(p\) of being selected next, then is it actually selected (by the human participant) this often?

We will calculate this as follows: for each target selection (on each trial, for each participant) we look at the weight assigned to the most likely item, and then look at whether it was selected or not.

```
my_breaks <- seq(0.0, 1, 0.05)

a %>% mutate(b_bin = cut(max_b, breaks = my_breaks, labels = FALSE)) %>%
  group_by(condition, b_bin) %>% 
  summarise(acc = mean(selected_max), .groups = "drop") %>%
  mutate(b_bin = as.numeric(b_bin)/(length(my_breaks)-1)) -> a_cut
```

We can see from Figure 2 below that our model is well-calibrated (at least in terms of the target with the highest assigned weight). If we repeat this calculation for the 2nd and 3rd ranked candidate items we can see that we slightly underweight these items, but we are still pretty accurate.

Figure 2: (*left*) Calibration plot for our foraging model. The *x*-axis gives the largest weight assigned by the model while the *y*-axis shows how often that target was actually selected by a human participant. (*right*) This plot shows how often the 2nd and 3rd ranked items are selected based on the weights assigned by the model.

## 1.2 Individual Differences

How often does each participant select the target with the largest weight?

```
a_agg %>% group_by(observer, condition) %>%
  summarise(accuracy = mean(prop_best), .groups = "drop") -> a_acc

ggplot(a_acc, aes(x= condition, y = accuracy, fill = condition)) + 
  geom_boxplot() +
  geom_line(aes(group = observer), alpha = 0.25, colour = "sienna4") +
  scale_y_continuous("model accuracy",  
                     limits = c(0.35, 0.8), breaks = seq(0.3, 0.8, 0.1)) + 
  theme(legend.position="none")-> plt_a

plt_a
```

Figure 3: Prediction scores for participants. Boxplots show quartile range and the grey lines indicate individual participants. (The dots indicate outliers.)

Is this explained by differences in proximity weighting? i.e., are participants with weaker proximity biases harder to predict? Yes!

Figure 4: Accuracy of our model varies with the strength of an individual’s model parameters. We can see two clear outlier participants (marked with an X).

As the two outlier participants appear to be doing something different, not captured especially well by our model, we will remove them from the rest of this analysis.

After removing them, we can see that individual differences in proximity tuning account for nearly all of the differences in predictability from person to person, and also between the *feature* and *conjunction* conditions (see Figure 4).

```
dm <- filter(fit, is_outlier == 0)

summary(lm(accuracy ~ condition * bP, 
           data = dm))
```

```
## 
## Call:
## lm(formula = accuracy ~ condition * bP, data = dm)
## 
## Residuals:
##      Min       1Q   Median       3Q      Max 
## -0.06036 -0.01560 -0.00022  0.01438  0.07154 
## 
## Coefficients:
##                          Estimate Std. Error t value Pr(>|t|)    
## (Intercept)              0.474140   0.017637   26.88   <2e-16 ***
## conditionconjunction     0.008716   0.027523    0.32     0.75    
## bP                       0.009342   0.000802   11.65   <2e-16 ***
## conditionconjunction:bP -0.001849   0.001594   -1.16     0.25    
## ---
## Signif. codes:  0 '***' 0.001 '**' 0.01 '*' 0.05 '.' 0.1 ' ' 1
## 
## Residual standard error: 0.0232 on 108 degrees of freedom
## Multiple R-squared:  0.822,  Adjusted R-squared:  0.817 
## F-statistic:  166 on 3 and 108 DF,  p-value: <2e-16
```

We can see that the difference in the proximity parameter between conditions appears to account for most of the variables in our model’s accuracy from one participant to the next.

We can also see if the other parameters have an effect.

```
summary(lm(accuracy ~ pA + pS + bM + bP, 
           data = dm))
```

```
## 
## Call:
## lm(formula = accuracy ~ pA + pS + bM + bP, data = dm)
## 
## Residuals:
##     Min      1Q  Median      3Q     Max 
## -0.0447 -0.0147 -0.0012  0.0124  0.0656 
## 
## Coefficients:
##              Estimate Std. Error t value Pr(>|t|)    
## (Intercept)  0.463123   0.026882   17.23  < 2e-16 ***
## pA           0.028592   0.012395    2.31    0.023 *  
## pS          -0.032859   0.019892   -1.65    0.101    
## bM           0.022293   0.003850    5.79  7.1e-08 ***
## bP           0.011276   0.000734   15.37  < 2e-16 ***
## ---
## Signif. codes:  0 '***' 0.001 '**' 0.01 '*' 0.05 '.' 0.1 ' ' 1
## 
## Residual standard error: 0.0205 on 107 degrees of freedom
## Multiple R-squared:  0.861,  Adjusted R-squared:  0.856 
## F-statistic:  166 on 4 and 107 DF,  p-value: <2e-16
```

Both \(p\_A\) and \(b\_M\) also have an effect on our model’s accuracy, but note there is only a small increases in \(R^2\) from just using \(b\_P\).

## 1.3 The relationship between time and model accuracy

## 1.4 Shorter trials are more predictable

It seems like shorter trials are easier for the model to predict, in both the feature and conjunction case (though the relationship appears to be clearer in the feature condition).

|  | Estimate | Est.Error | Q2.5 | Q97.5 |
| --- | --- | --- | --- | --- |
| conditionconjunction | 0.598 | 0.005 | 0.588 | 0.608 |
| conditionfeature | 0.660 | 0.008 | 0.645 | 0.674 |
| conditionconjunction:time\_taken\_s | -0.008 | 0.004 | -0.016 | 0.000 |
| conditionfeature:time\_taken\_s | -0.022 | 0.005 | -0.032 | -0.012 |

This relationship appears to hold true if you look at how long it takes participants to select individual targets as well (Figure 5).

Figure 5: The relationship between the time taken to complete a single target selection and how predictable it is.

## 1.5 Examples of Trials

Let’s score every trial by how well the model captures behaviour (Figure 6), and then plot some different examples.

```
a %>% filter(!(observer %in% c(35, 45))) %>%
  group_by(observer, condition, trial) %>% 
  summarise(prop_max = mean(selected_max), .groups = "drop") %>% 
  arrange(desc(prop_max)) %>%
  ungroup() -> a_trl
```

Figure 6: Histogram of how predictiable individual trials are, defined as how often the model assigned the largest weight to the target that was then selected.

Let’s plot some examples of highly predictable (top 5%), typical (middle 10%) and unpredictable (bottom 5%) trials.

```
qs <- quantile(a_trl$prop_max, c(0.05, 0.40, 0.60, 0.95))

trls_hard <- filter(a_trl, prop_max < qs[1]) %>% sample_n(3)
trls_mid  <- filter(a_trl, prop_max > qs[2], prop_max < qs[3]) %>% sample_n(3)
trls_easy <- filter(a_trl, prop_max > qs[4]) %>% sample_n(3)
```

We can see that for these highly predictable trials (Figure 7), we make very few mistakes, and those mistakes that we do make appear to be related to some sort of shortest-path optimization on the part of the participant.

Figure 7: A random selection of *good* trials. When participants diverge from the model’s prediction, it appears to be due to some form of path-length optimisation.

Here are some typical trials (Figure 8). Possible we have the path-length minimization issue again.

Figure 8: A random selection of *typical* trials. Along with path-minimising behaviour, we can also see some disagreement around switching item type.

Now let’s look at less predictable (bottom 5%-tile) trials (Figure 9). Even here, the model does a relatively good job.

Figure 9: A random selection of *hard* trials. It is harder to make out much of a pattern here, but we can see that the human participants jump around more.

# 2 Path Length Analysis

It looks like some of the target selections that our model fails to capture are when participants select a further away target in order to minimise overall path length. Here we will compare our model’s predicted path length against the empirical paths.

We will provide the model/simulation with the initial target selection.

Figure 10: (*left*) Histogram comparing the empirical path lengths to simulations from our fitted model. Difference indicates the human path length - the simulated path length. (*right*) Histogram comparing the median path lengths per participant.

We can see that overall, the model is not making substantially longer paths through the items than our human participants (Figure 10). However, there is some variability in that some participants routinely select paths shorter than our model would, and others do not.

Perhaps this is related to predictability (i.e., how well the model can predict a participant’s trial level behaviour - Figure 11)?

Figure 11: (*left*) A histogram showing the proportion of trials on which each participant managed a shorter path through the targets than the model’s prediction. (*right*) Scatter plot showing the relationship between predictability (accuracy) and whether the model over or under estimates path length. Xs mark the outlier participants discussed above.

# 3 Initial Selection

Previous work (Clarke and Tatler, 2014) has demonstrated that the initial fixation in scene viewing can be well described with truncated Gaussian distribution. Here we explore using a similar idea to account for starting locations (Figure 12).

```
# Reimport data again (as we removed the initial target selection earlier)
d <- read_csv("data/clarke_2020_qjep.csv", show_col_types = FALSE) %>%
  filter(found == 1) %>%
  mutate(condition = as_factor(condition))
```

Figure 12: (*left*) Density plot for initial target selections, over all participants and all trials. (*right*) Median \(x\) and \(y\) coordinates for the initial target selection for each participant.

## 3.1 Multi-level Model

Can we model this as multi-level beta distribution? Yes, although it seems like people are in one of two clusters (Figure 13).

Figure 13: The mean posterior plot for the truncated normal distriubtions for (*left*) \(x\) and (*right*) \(y\). We can see that a two component mixture model will capture most of the variance in intial target selection.

## 3.2 Mixture Model

It looks like we can simplify the model somewhat by using a two component mixture model instead (Figure 14).

Figure 14: (*top*) Beta distributions for \(x\) and \(y\) coordinated

## 3.3 Posterior Predictions

Both models give a similar distribution of target weights and accuracies, and in both cases, participants who start in a corner are easier to predict (Figure 15).

Figure 15:(Top): a count of initial weight values predicted from both methods (mixture and multi-level). (Bottom left): the proportion of times the first selection was correct for both methods. (Bottom right): as lambda (the model parameter underlying initial weight) increases, accuracy increases ie. the model is moore accurate for the cases where participants are selecting a corner as their initial target selection.

| method | mean\_score | mean\_accuracy |
| --- | --- | --- |
| mixture model | 0.215 | 0.399 |
| multi-level | 0.181 | 0.382 |
