# Supplementary material for: A Bayesian Statistical Model Is Able to Predict Target-by-Target Selection Behaviour in a Human Foraging Task
Source: Vision (Basel). 2022 Nov 11;6(4):66. doi: 10.3390/vision6040066 (PMC9680426; doi:10.3390/vision6040066)
Supplement: Supplementary file 1 [file vision-06-00066-s001.zip › supplementary materials part 2.html]

SVG: The Paper (2nd part of supplementary material)


# SVG: The Paper (2nd part of supplementary material)

#### Alasdair Clarke, Amelia Hunt & Anna Hughes

#### 14/10/2022

Terminology questions: - item v target - participant v observer

# 1 Trial Level Posterior Predictions

This is the replication with Kristjansson (2014) data.

```
# read in data
d <- read_csv("data/test_arni.csv", show_col_types = FALSE) %>%
  mutate(condition = as_factor(condition),
         condition = fct_recode(condition, feature = "1", conjunction = "2"),
         targ_type = as_factor(targ_type))
```

Here, we have used half the data as a training set which we used to
fit the initial model. This is then used for computing model weights for
the test dataset.

For simplicity’s sake, we will characterise all of our estimated
posterior probability densities with just the mean value. This gives us
four parameters per participant: \(b\_S, b\_A,
b\_P, b\_D\).

Using these fitted values, we can now step through each target
selection in the dataset and computer the probability weights our model
assigns to each remaining target. For each target selection, we
record:

- the item assigned the highest weight by our model.
- whether this item was then selected by the human participant.

```
# item weights pre-computed - see scripts/extract_item_weights_using_model.R
a <- readRDS("scratch/kristjansson_model_weights_train.rda") %>%
  filter(found != 1) # remove initial selections

# compute the average weight for each participant x condition x item selection
# also compute the proportion of times in which the item selected by the 
# participant was judged the most likely to be selected by our model.
a %>% group_by(condition, observer, found) %>%
  summarise(meanb = mean(b),
            prop_best = mean(selected_max), .groups = "drop") %>%
  # finally, also compute what we would expect under a null-model
  mutate(chance = 1/(41-found))-> a_agg
```

We can then plot this data to show how our ability to predict which
target will be selected next varies throughout a trial (Figure 1). We
can see a difference between *feature* and *conjunction*
foraging, with *feature* foraging being more predictable (though
the distinction is less clear after the initial 20 target
selections).

Figure 1: (*left*) The average weight assigned to each selected
target by our model. (*right*) The proportion of trials the item
with the largest assigned weight was selected by the participant. Each
dot shows data from an individual participant in a condition and the
shaded region indicates the interval in which we expect 67% of
participants to fall.

## 1.1 Calibration

Is our model calibrated? By this we mean, if our model assigns an
item a probability \(p\) of being
selected next, then is it actually selected (by the human participant)
this often?

We will calculate this as follows: for each target selection (on each
trial, for each participant) we look at the weight assigned to the most
likely item, and then look at whether it was selected or not.

```
my_breaks <- seq(0.0, 1, 0.05)

a %>% mutate(b_bin = cut(max_b, breaks = my_breaks, labels = FALSE)) %>%
  group_by(condition, b_bin) %>% 
  summarise(acc = mean(selected_max), .groups = "drop") %>%
  mutate(b_bin = as.numeric(b_bin)/(length(my_breaks)-1)) -> a_cut
```

We can see from Figure 2 below that our model is well-calibrated (at
least in terms of the target with the highest assigned weight). If we
repeat this calculation for the 2nd and 3rd ranked candidate items we
can see that we slightly underweight these items, but we are still
pretty accurate.

Figure 2: (*left*) Calibration plot for our foraging model. The
*x*-axis gives the largest weight assigned by the model while the
*y*-axis shows how often that target was actually selected by a
human participant. (*right*) This plot shows how often the 2nd
and 3rd ranked items are selected based on the weights assigned by the
model.

### 1.1.1 Individual Differences

How often does each participant select the target with the largest
weight?

```
a_agg %>% group_by(observer, condition) %>%
  summarise(accuracy = mean(prop_best), .groups = "drop") -> a_acc

ggplot(a_acc, aes(x= condition, y = accuracy, fill = condition)) + 
  geom_boxplot() +
  geom_line(aes(group = observer), alpha = 0.25, colour = "sienna4") +
  scale_y_continuous("model accuracy",  
                     limits = c(0.35, 0.8), breaks = seq(0.3, 0.8, 0.1)) -> plt_a

plt_a
```

Figure 3: Prediction scores for participants. Boxplots show quartile
range and the grey lines indicate individual participants. (The dots
indicate outliers.)

Figure 3 shows that model accuracy is quite high (though lower than
for Clarke et al).

Is this explained by differences in proximity weighting? i.e., are
participants with weaker proximity biases harder to predict? Yes (Figure
4).

Figure 4: Accuracy of our model varies with the strength of an
individual’s model parameters. We can see two clear outlier participants
(marked with an X).

We can see that individual differences in proximity tuning account
for nearly all of the differences in predictability from person to
person, and also between the *feature* and *conjunction*
conditions.

```
dm <- filter(fit, is_outlier == 0) # have not currently defined anything as an outlier

summary(lm(accuracy ~ condition * bP, 
           data = dm))
```

```
## 
## Call:
## lm(formula = accuracy ~ condition * bP, data = dm)
## 
## Residuals:
##      Min       1Q   Median       3Q      Max 
## -0.09824 -0.02802 -0.00433  0.03042  0.08559 
## 
## Coefficients:
##                         Estimate Std. Error t value Pr(>|t|)    
## (Intercept)              0.13330    0.06596    2.02    0.053 .  
## conditionconjunction     0.46728    0.19164    2.44    0.021 *  
## bP                       0.01844    0.00264    6.97  1.4e-07 ***
## conditionconjunction:bP -0.02152    0.00973   -2.21    0.035 *  
## ---
## Signif. codes:  0 '***' 0.001 '**' 0.01 '*' 0.05 '.' 0.1 ' ' 1
## 
## Residual standard error: 0.0454 on 28 degrees of freedom
## Multiple R-squared:  0.668,  Adjusted R-squared:  0.633 
## F-statistic: 18.8 on 3 and 28 DF,  p-value: 7e-07
```

We can see that the difference in the proximity parameter between
conditions appears to account for most of the variables in our model’s
accuracy from one participant to the next.

We can also see if the other parameters have an effect - which they
do seem to.

```
summary(lm(accuracy ~ pA + pS + bM + bP, 
           data = dm))
```

```
## 
## Call:
## lm(formula = accuracy ~ pA + pS + bM + bP, data = dm)
## 
## Residuals:
##      Min       1Q   Median       3Q      Max 
## -0.05041 -0.02040 -0.00591  0.02775  0.05571 
## 
## Coefficients:
##             Estimate Std. Error t value Pr(>|t|)    
## (Intercept)  0.25903    0.06900    3.75  0.00085 ***
## pA           0.09687    0.04396    2.20  0.03626 *  
## pS           0.09003    0.04944    1.82  0.07968 .  
## bM           0.05228    0.01294    4.04  0.00040 ***
## bP           0.01336    0.00154    8.69  2.6e-09 ***
## ---
## Signif. codes:  0 '***' 0.001 '**' 0.01 '*' 0.05 '.' 0.1 ' ' 1
## 
## Residual standard error: 0.0318 on 27 degrees of freedom
## Multiple R-squared:  0.843,  Adjusted R-squared:  0.82 
## F-statistic: 36.2 on 4 and 27 DF,  p-value: 1.73e-10
```

# 2 Initial Selection

Similar to Clarke et al, there is a bias towards starting in a corner
(Figure 5). Interestingly, there isn’t much evidence of a central bias
(possibly because these trials were completed on an iPad).

```
# Reimport data again (as we removed the initial target selection earlier)
d <- read_csv("data/test_arni.csv", show_col_types = FALSE) %>%
  filter(found == 1) %>%
  mutate(condition = as_factor(condition))
```

Figure 5: (*left*) Density plot for initial target selections,
over all participants and all trials. (*right*) Median \(x\) and \(y\) coordinates for the initial target
selection for each participant.
